# Socio-Cultural Aspects of Chagas Disease: A Systematic Review of Qualitative Research

**DOI:** 10.1371/journal.pntd.0002410

**Published:** 2013-09-12

**Authors:** Laia Ventura-Garcia, Maria Roura, Christopher Pell, Elisabeth Posada, Joaquim Gascón, Edelweis Aldasoro, Jose Muñoz, Robert Pool

**Affiliations:** 1 Barcelona Centre for International Health Research (CRESIB, Hospital Clínic, Universitat de Barcelona), Barcelona, Spain; 2 Centre for Social Science and Global Health, University of Amsterdam, Amsterdam, The Netherlands; René Rachou Research Center, Brazil

## Abstract

**Background:**

Globally, more than 10 million people are infected with *Trypanosoma cruzi*, which causes about 20 000 annual deaths. Although Chagas disease is endemic to certain regions of Latin America, migratory flows have enabled its expansion into areas where it was previously unknown. Economic, social and cultural factors play a significant role in its presence and perpetuation. This systematic review aims to provide a comprehensive overview of qualitative research on Chagas disease, both in endemic and non-endemic countries.

**Methodology/Principal Findings:**

Searches were carried out in ten databases, and the bibliographies of retrieved studies were examined. Data from thirty-three identified studies were extracted, and findings were analyzed and synthesized along key themes. Themes identified for endemic countries included: socio-structural determinants of Chagas disease; health practices; biomedical conceptions of Chagas disease; patient's experience; and institutional strategies adopted. Concerning non-endemic countries, identified issues related to access to health services and health seeking.

**Conclusions:**

The emergence and perpetuation of Chagas disease depends largely on socio-cultural aspects influencing health. As most interventions do not address the clinical, environmental, social and cultural aspects jointly, an explicitly multidimensional approach, incorporating the experiences of those affected is a potential tool for the development of long-term successful programs. Further research is needed to evaluate this approach.

## Author Summary

Social and cultural factors are increasingly recognized as relevant to the likelihood of infection with Chagas disease as well as the health seeking practices of those affected. In response, qualitative methods have been used more often to study such factors. This is the first systematic review to focus on the socio-cultural dimensions of Chagas disease. The findings suggest that transdisciplinary approaches, which incorporate preventive and treatment activities, and consider populations' living conditions and their culturally informed understandings of health, might be a tool to reduce Chagas disease incidence in the long-term.

## Introduction

According to the World Health Organization (WHO), globally, more than 10 million people are infected with *Trypanosoma cruzi*. Most live in Latin American countries, where the parasite is endemic [1] and where control strategies were first implemented in the 1940s and 1950s [2]–[3]. Since 2000, due to expanding migration flows and increased funding for research on Neglected Tropical Diseases (NTD), Chagas has become an international health priority. Estimates suggest that between 50,000 and 70,000 are affected in Spain [4], and 300,000 in the USA [5].

The persistence of Chagas disease – as with most NTD – is linked to social, cultural, historical, political and economic processes [6]–[8]. The WHO recognizes Chagas disease as one of the most neglected diseases, mainly found amongst the poorest [9]. Within countries, its unequal distribution illustrates the complex interaction of socio-cultural, biological and environmental factors. The creation of the WHO's Special Programme for Research and Training in Tropical Diseases (TDR) in 1975 saw the social sciences incorporated into the study of NTD [6], [10], [11]. However, a recent bibliographic analysis [7] has shown that social science contributions remain scarce. Hence there is a paradox: although the importance of social and cultural factors is broadly acknowledged, current approaches to NTDs almost always neglect aspects of the socio-cultural - biologically - environment triad [10]. This results in a narrower understanding of Chagas disease and hampers sustainable prevention and control [6].

Since 1990, the number of Chagas-related publications has shown a linear increase [3], [12]. What has qualitative social science research contributed? What are the main research gaps? What are next steps for future research? Under the auspices of the COHEMI project and as part of *Work Package 6: Social and Cultural Context to Health Seeking of Latin American Migrants in Europe*, this systematic review aims to provide a comprehensive overview of qualitative research on Chagas disease, both in endemic and non-endemic countries. Specific objectives include identifying qualitative studies through a systematic search strategy; describing the state of qualitative research; and synthesizing, analyzing and interpreting themes that have emerged [13]–[14]. This enables the identification of research gaps, contradictory findings and priorities for further research. The review is restricted to English, Spanish and Portuguese language literature. No previous literature review on this topic has been identified.

## Methods

### Search strategy

Literature was identified using a search strategy available at www.cohemi-project.eu. Ten databases were examined using combinations of terms (see Table 1), and other sources of information, such as the bibliographies of identified articles, were used. The most recent searches were carried out in November 2012.

**Table 1:** List of databases and search terms.

DATABASE	CONCEPTS	
Embase 1980 to 2012 week14, Ovid MEDLINE 1946 TO MARCH Week 4 2012, Social policy and practice 201201	Chagas.m_titl., *Trypanosoma cruzi*.m_titl., *T.cruzi*.m_titl. (Limit to human (not valid in Social Policy and Practice))	qualitative research.af., anthropology.af., ethnology.af., ethnography.af., social sciences.af., beliefs.m_titl., health seeking.m_titl., experienc$.m_titl., practices.m_titl., representations.m_titl., behavior.m_titl
LILACS	Chagas, *Trypanosoma cruzi*	qualitative, anthropology, ethnography, qualitative research, psychology, social sciences
JSTOR	ti:(Chagas, *Trypanosoma cruzi, T.cruzi*)	
Scopus	Chagas, *Trypanosoma cruzi*, trypanosomiasis (NOT Africa), *T.cruzi*. (Limit to human)	qualitative research, ethnography, ethnology, social sciences, anthropology (all fields)
Crochane Plus	Chagas, *Trypanosoma cruzi, T.cruzi*	qualitative, anthropology, social sciences
TESEO	(Chagas, *T.cruzi, Trypanosoma cruzi*):ti and abstract	
IME-ISOC (CSIC)	Chagas, *Trypanosoma cruzi, T.cruzi*	

**Figure 1:**
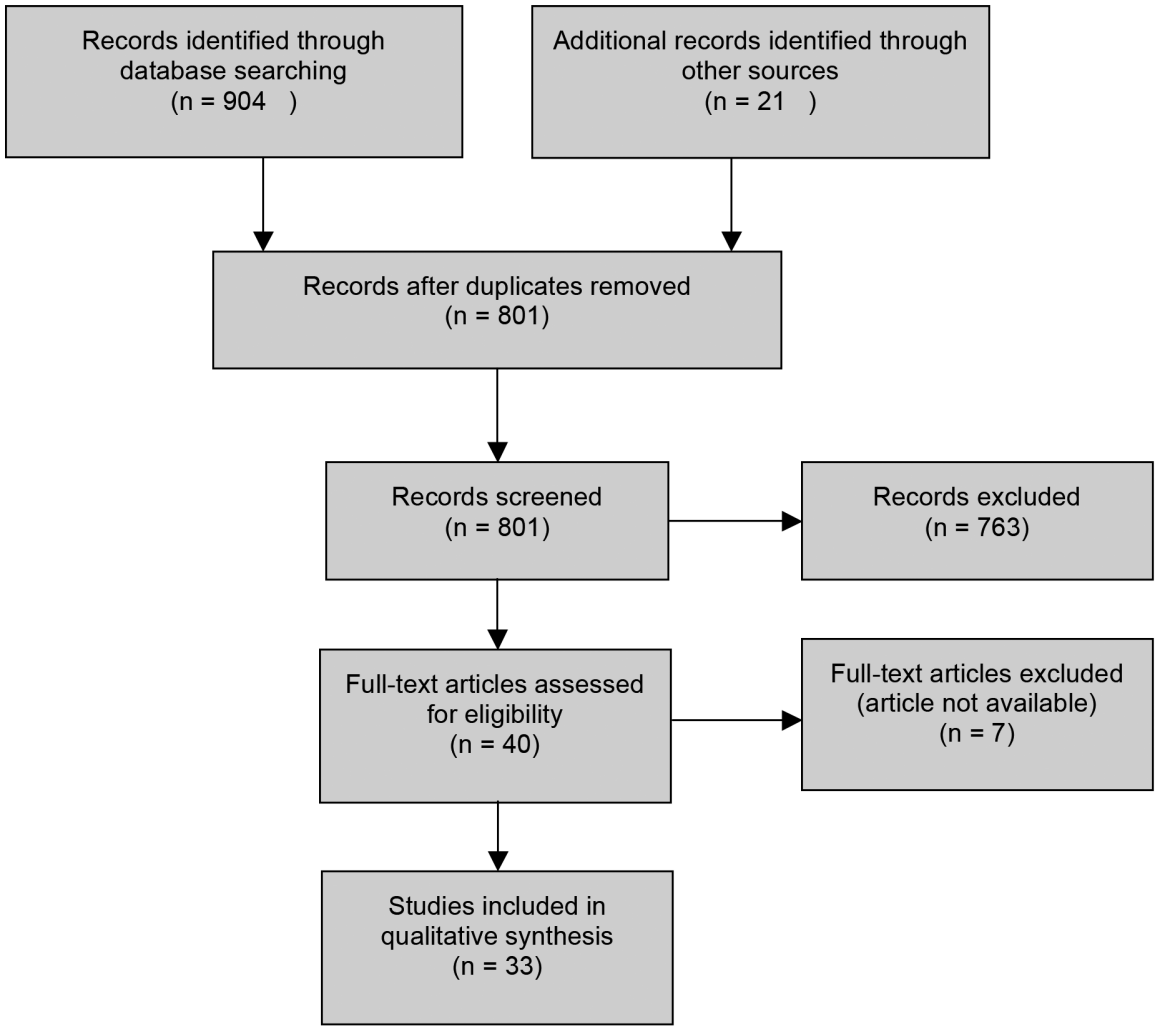
Flow of search.

Citations and abstracts were downloaded into Mendeley and Endnote 6 by two independent researchers, and duplicates were deleted. A preliminary screening of titles and abstracts was performed according to the following inclusion criteria: research related to Chagas disease; employed qualitative methods and findings were derived from qualitative methods; English, Spanish and Portuguese language. Methods considered to be qualitative were interviews, observation or participant observation, focus groups, ethnography, discourse analysis and participatory methods. Of the articles that reported results based on quantitative and qualitative methods, only the latter data were reviewed. Surveys and book reviews were not included. Access to the full text of the remaining articles was sought; seven articles could not be accessed (see Figure 1).

### Analysis

A thematic synthesis approach was taken to analyze the literature [13], [14]. Software for qualitative data analysis –AtlasTi– was used to code selected documents. “Descriptive themes” that resulted from this process enabled the generation of “analytical themes”, as a next step of interpretation. Articles were classified by fieldwork site and main topic (see Appendix S1 and Appendix S2).

## Results

### Search results

Of the thirty-three studies reviewed, the majority (27) were conducted in Latin America: Argentina (12), Brazil (7), Bolivia (2), Peru (2), Venezuela (2), Paraguay (1) and Colombia (1). Of the twelve studies carried out in Argentina, half were by the same author; this was also the case for two articles from Brazil. In non-endemic countries, studies were conducted in Europe: Spain (4) and Italy (1); and one in USA.

Most of the studies were based on individual interviews (24). In ten cases, this was the only reported technique for collecting data; twelve articles reported the complementary use of either: individual interviews and participant observation (7); individual and group interviews (2); individual interviews and participatory tools (1); individual interviews and other techniques (2); in three articles, researchers utilized more than two techniques. Finally, four articles reported participatory methods; discourse analysis techniques (2); and focus groups (2). Most of the articles have been published since 2008 (See Table 2).

**Table 2:** Year of publication of the articles reviewed.

Year published	N
2008–2012	16
2003–2007	7
1998–2002	9
1993–1997	1

### Qualitative synthesis of findings: Endemic countries

#### The interplay of socio-structural factors and Chagas disease

Chagas disease occurs in specific contexts marked by socio-cultural, political, economic, environmental and historical circumstances. Certain structural changes may influence the environment and people's living conditions, which, in turn, may trigger triatomin infestation (the Chagas disease insect vector) and provoke a higher risk of infection. Briceño-León [15] and Mastrangelo [16] described such case in the Amazon Region and Argentina, respectively: a shift in economic development during the 1980s towards industrial production and international trade played an important role determining social and environmental changes, which led to deterioration in living conditions. Land expropriations and deforestation forced families to migrate and engage in wage labor. Triatomins, previously located in more wooded areas, began occupying domestic spaces; and household re-infestation increased, in part because of its precarity.

Several reviewed studies focused on migration and stigmatization of infected people as pathways that caused their living conditions to deteriorate. Bayer et al. [17] showed how, in neighboring communities in Peru, vulnerability to triatomine infestation depended on the socio-economic processes underlying migration and settlement patterns. Displacements from endemic rural areas to urban settings or from non-endemic areas to endemic areas - for permanent or temporal labor - contributed to the appearance of infections in urban and non-endemic rural areas [16]–[20]. Other studies explored how the stigmatization of poor and rural populations and the discrimination of infected individuals affected their access to health-care and working conditions. Studies in different parts of Argentina [16], [21]–[22] described the mistreatment and discrimination that ethnic groups experienced in medical contexts, limiting their access to care. Employment-related stigma and discrimination [18], [23]–[24] was highlighted in urban settings [18], [23], [25]–[26], where Chagas disease - even positive serology regardless of symptoms – often resulted in labor exclusion. Chagas sufferers therefore commonly avoided the diagnostic tests that were often a prerequisite for employment, and sought informal sector, which caused deteriorations in living conditions. Chagas-related stigma also affected more socially and economically favored groups [18], [26]–[27]. Chagas disease's association with filth, neglect and misery led such groups to ignore possible infections, avoid testing, and meant that positive diagnosis caused great suffering.

#### Health practices

Hygienic habits, household clutter, or cohabitation with domesticated animals were often identified as behaviors influencing Chagas prevention, and the persistence of the triatomine vector in dwellings [16], [18]–[19], [21]–[22], [25]–[26], [28]–[32]. Caballero Zamora et al. [28] and Rojas et al. [29] explained these behaviors in terms of a lack of knowledge about Chagas disease and its transmission. Therefore, as Sanmartino et al. [30] described, greater knowledge of Chagas disease would drive communities to acquire new health-related behaviors.

However, this was not always the case, and other authors, including Sanmartino in subsequent research, suggested that increased knowledge of Chagas disease was not always enough to change practices [16], [18]–[19], [25], [28]: “risk perceptions” [16], [18]–[19], [25], [28], and the ways of thinking about Chagas within a population's worldview [16], [18], [21]–[22], [25]–[26], [31]–[32] might explain behaviors. Five studies [16], [18]–[19], [25], [28] suggested that community members' views about the danger of triatomins and Chagas disease often differed from those of policy makers and clinicians. For example, in some highly endemic areas, where living conditions were demanding and the vector very common, Chagas disease was not perceived as a threat or, at least, as a health priority. Therefore, behavior was not always researchers expected. Moreover, an absence of symptoms and of an impact on everyday activities contributed to the *naturalization* and *normalization* of Chagas disease [25]–[26], [31], which influenced care seeking [19].

Insight into different ways of thinking about Chagas disease, its *socio-cultural representations*, was however seen as crucial to understand behavior and explain why knowledge about Chagas disease does not always imply behavior change [16], [18], [21]–[22], [25]–[26], [31]–[32]. For example, taking into account the failure of previous preventive programs based on increasing knowledge amongst indigenous populations in Las Lomitas, Argentina, Sosa-Estani [22] highlighted how, from the indigenous (*pilagá*) point of view, disease causation was explained in personalistic terms, or as violation of taboos or social norms. Preventive and curative practices may be therefore deeply linked to such representations, rather than the parasite or insect-vector. In Bolivia [28], some indigenous understanding of the vector as bearers of good luck was also a way to explain inaction against triatomines. In “peri-domestic” settings of Argentina's Gran Chaco region, the failures of vector control activities were attributed to differences between the conceptions of “landscape”, “wild” and “domesticated” used in preventive programs and those of the target population [16]. Preventive programs differentiated “domestic” from “peri-domestic” spaces as an indicator of human proximity to the insect vector, whereas the local population conceived these spaces as a continuum, allowing humans, animals and plant material brought from the mountain to cohabit “domestic” spaces.

#### Biomedical understandings of Chagas disease

Particular ways of thinking about Chagas disease are not unique to target populations. Scientists' and health professionals' understandings are also developed in specific contexts and can affect their work. Kreimer et al. [3] reflected historically on the production of scientific knowledge on Chagas disease in Argentina: the “problem of Chagas” was defined according to the interests of different stakeholders, which involved recognizing certain facts and neglecting others. Historically, revised definitions of appropriate intervention strategies have also involved re-conceptualizations of Chagas disease: first seen as a problem of precarious living conditions; then as a question of fumigation; and finally, as a matter for basic research, for which molecular biology was prioritized. Ramos et al. [33] addressed the role of Brazilian mass media in the construction of the problem: positive assessments of Chagas' control strategies contributed to the social demobilization of a problem that was not yet under control.

Addressing health professionals' understandings of Chagas disease in an Argentinean urban context and in Brazil, Sanmartino [34] and Uchoa et al. [24] highlighted how a lack of relevant training and their own stereotypes affected the care they provided.

#### The patient's experience of Chagas disease

Studies that addressed experiences of Chagas disease from the sufferer's perspective showed how social groups described a variety of conceptions about the disease [21], [24], [35]–[37]: local communities do not only have knowledge and specific ways of thinking about “Chagas”, but people also syncretize different kinds of knowledge, producing new hybridized understandings.

Evaluating the first Chagas' preventive programs in Brazil, Magnani et al. [35] concluded that the emphasis placed on disease contributed to a rapid and effective response: it provoked awareness of people's responsibility for health and their role in combating the vector, and led to new preventive practices. Nevertheless, prevention programs that neglected those already affected disregarded the meanings attributed to “Chagas”: new meanings, such as associations with death, fear, suffering, distrust, and despair, caused suffering and could affect health seeking behaviors. For example, two studies from Brazil [24], [35] described how some people developed informal yet adaptive strategies, such as denying Chagas disease, when the emotional burden was unbearable.

In contrast, studies in Brazil [35] and Argentina [18] described how a positive serology can be enough to consider oneself as ill, even if asymptomatic: although biomedical nosologies classify different degrees of severity of Chagas disease based on symptoms, infection by *Trypanosoma* was conflated with diagnosis of Chagas disease. A positive serology saw infected individuals identify themselves as sick, was sufficient to promote feelings of discomfort, and caused individual and social suffering. In all these situations, the patient-provider relationship was identified a key element in patients making sense of the disease [16], [18], [24], [35]–[36].

#### Institutional strategies

There is no effective vaccine for Chagas disease and treatments are not 100% effective (in chronic cases effectiveness is unknown, although known to be low). Therefore, in endemic countries, the main strategies to combat the disease involve prevention interventions, control of blood banks and monitoring pregnant women. Preventive strategies have focused on controlling the most common vector through three principal interventions [25]: housing improvements, insecticide spraying, and health education. Many of the reviewed studies were designed to supplement, improve or evaluate Chagas prevention and/or control interventions [16], [18]–[20], [22], [25], [27], [28]–[32], [34]–[40]

Four studies [16], [18], [24], [35] highlighted the need for these approaches, but raised the question of whether achievements in vector control led to improvements in social and individual wellbeing. Surveillance and control activities were often dissociated from diagnosis, management and care. Moreover, because Chagas disease was problematized with reference to a narrow biological framing that neglected other political, economical, socio-cultural and individual dimensions, activities focused on infected individuals or those at risk of infection were neglected.

### Qualitative synthesis of findings: Non-endemic countries

#### Access to health services

In non-endemic countries, Chagas control is based on screening protocols for migrants considered at risk of infection. Several studies highlighted the importance of monitoring migrants' access and utilization of health services [41]–[45]. In Europe, migrants' lack of information about services and working constraints were the main barriers to accessing health services [41]–[45]. Immigration policies [41], migrants' mobility and Chagas disease's lack of symptoms [42] were also highlighted. Although one study [42] concluded that a “lack of knowledge” about Chagas disease prevented Latin American migrants from accessing screening services in Madrid, an ethnography conducted in Barcelona [43] revealed that migrants tend to feign ignorance during medical consultation - waiting for information from health personnel - and discuss their experiences and knowledge in other contexts. Although the author highlighted the importance of providing information about Chagas disease, associated fears and meanings explained why some migrants resisted screening. Indeed, meanings of Chagas disease and experiences within the health system in migrants' countries of origin were key to understanding health-seeking in Spain. In USA [45], a lack of awareness about Chagas disease among Latinos and fears of being diagnosed with a disease associated with death were also reported as barriers to testing and treatment, together with reservations about quality of care and costs.

Gender relationships and identity also influenced migrants' patterns of healthcare utilization: men were less likely to utilize healthcare services because they associated seeking help with weakness, which challenged their ideals of masculinity. Furthermore, due to their social role as breadwinners, men prioritized their families' economic situation over their own health- [43]–[44]. Indeed, men did not use to seek care until symptoms were unbearable [45]. In contrast, women had closer ties to the health system due to their reproductive role. Mothers' feelings of responsibility and guilt, if their children received a positive diagnosis, led them to use health services to ensure their children received treatment [43]–[45].

Studies emphasized the importance of developing community-oriented programs [41]–[43], as well as the need to consider the wider social determinants of health-care access services and health-related – such as the factors that limit patients' capacity to alter their dietary habits to reduce Chagas-related constipation [46] - into strategies and health recommendations [41], [46].

#### Migratory goals as incentives for seeking care

A study conducted in Madrid [44] described how Bolivian migrants attended a specialized tropical disease unit with chronic symptomatic Chagas disease, and following the death or diagnosis of a friend or relative. Patients were referred by family, friends, or through primary health care facilities. However, migrants' main motivations for seeking care were not only associated with the somatic dimension of Chagas disease; patients' main concerns were linked to their life projects and dying before achieving their migratory goals: ensuring the welfare of relatives, who depended on their income and the health of their children, was essential [43]–[44]. Although social relations, the family and migratory projects were key to understanding health seeking, these motivations contrasted with a disease-centered approach utilized in control interventions and care programs. This contrast was complicated by the mostly asymptomatic nature of the disease, its association with death and severe illness, and the lack of cure or effective treatment.

Regarding treatment, some patients claimed that it could lengthen life, and hence was a guarantee of achieving migratory goals [43]–[44]. Other asymptomatic patients considered it an unnecessary risk, assuming that symptoms would only present after achieving their migratory goals [44]. Side effects and uncertain outcome were reasons for refusing or abandonment treatment.

## Discussion

As the work of Bastien [47] and Briceño-León [48] suggest, the literature reviewed illustrates how Chagas disease is embedded in a web of relationships marked by biological, socio-cultural, political, economical, historical and environmental circumstances that shape its incidence and prevalence, as well as population's response.

The reviewed articles identified pathways that lead certain social groups into conditions that increase their vulnerability to Chagas disease [15]–[26], [31]. The impacts of macro-economic policies on local communities, national and international economic/labor migration, or the social and political exclusion of poor, rural or ethnic groups were some of the processes described. Nonetheless, wide ranges of social determinants that foster propitious conditions for infection and prevent social group from changing their behavior have been mostly neglected in Chagas disease control strategies. Gaining insights into the processes that increase vulnerability to infection in specific contexts is however fundamental to orient and adapt interventions to local settings. Such an understanding can assist the development of national and international policies and the implementation of the most suitable prevention measures in a given context.

With regard to prevention and control activities, behavioral change approaches at an individual and community level have had varying degrees of success. In the reviewed literature, there were two general approaches. In one approach a population's lack of knowledge and its members' beliefs were assumed to be “cultural obstacles” to behavior change or to care seeking [28]–[30], [42]. A second approach however proposed an alternative: knowledge is not enough to change behavior, and populations' understanding of health and Chagas disease - consistent or not with biomedical models - and living conditions play an important role with regard to behavior [16], [18]–[19], [21]–[22], [25]–[26], [28], [31]–[32], [43]–[44].

The reviewed highlights an important question: how can awareness about Chagas disease and triatomines be raised in social groups that do not conceive them as a health threat? Scholars have suggested that people's understandings of Chagas and the social context that influences their living conditions are key to explain the disease not being considered a threat [16], [18]–[19], [21]–[22], [25]–[28], [31]–[32], [43]–[44]. When living conditions are demanding, as is common in endemic rural areas, Chagas disease is not often a priority. However, socially and economically favored groups do not often consider the possibility of infection. Inversely, when Chagas disease is perceived to be a problem, reasons may not be linked to the disease itself, but to its social impact, such as hindering migratory goals or its financial affects on a patient's children. Prevention and care practices are often therefore less disease-specific than clinicians and policy makers' understandings. Moreover, understanding how these processes vary across social groups is key to the design and implementation of appropriately adapted interventions.

In relation to local ways of thinking about Chagas disease, a second question arose about how to recognize local knowledge and build bridges with biomedicine [18]. People are not merely recipients of information and, although social groups handle Chagas-related knowledge and meanings based on their socio-cultural worldviews [16], [21], [24], [35]–[37], [44]–[45], these models are not closed and pure. Instead they are syncretic: for example in Brazil [37], where during early prevention programs, new conceptions of Chagas disease were formed based on pre-existing knowledge and the information disseminated. Chagas-related meanings can also change during migration, when they are re-orientated according to the new situation and possibilities [44]–[45]. Preventive interventions and doctor-patient interactions are spaces where new conceptions about Chagas disease are constructed. Inasmuch as people use to react to the meanings associated with Chagas disease and not just to the disease itself [44], messages transmitted in preventive programs, doctor-patient communication, and the coordination of Chagas prevention and control programs with disease management patient care, are fundamental. Transdisciplinary activities that consider the experience and needs of those affected might be a successful way of creating links between biomedical and local understandings, opening new possibilities for Chagas disease prevention and management.

### Priorities for further research

Further research is required to contextualize socio-cultural factors and processes associated with Chagas disease in different countries and amongst different social groups: differentiated by class, ethnicity, geography, gender and age. This necessitates the study of Chagas disease in relation to the socio-cultural, economical, political, historical processes that enable its appearance and persistence, as well as the different experiences of Chagas disease. Research concerning policies and programs to address the socio-structural factors is needed to reduce the burden of Chagas disease; understanding the elements that hinder the development and implementation of such policies is also important.

In most countries, there is a general lack of qualitative studies informing locally and nationally appropriate strategies. Contextualized research on migratory flows, how they interact with living conditions, and their links with Chagas incidence is necessary. In addition, research on changing risk behaviors for infection that takes into account the processes that place social groups in different positions of vulnerability is required. Such approaches are rare in research on and interventions for Chagas, though they may be crucial for policy and action guidance.

There is a lack of information concerning health seeking behaviors and their underlying processes in endemic countries. As studies described self-care practices [18], [24], [28], [35]–[36],[45], as well as deficiencies in diagnosis, care and treatment for Chagas disease, understanding the processes underlying the specific care seeking steps could contribute to the design and/or re-orientation of prevention, screening and care programs, both in endemic and non endemic countries. A deeper knowledge of experiences of an asymptomatic disease and its relationship with health seeking could also be helpful to understand health seeking processes.

Because Chagas disease is considered a zoonosis that cannot be eradicated, long-term entomological surveillance systems are required [49]. Further research on the effects of the organization of health systems, especially to clarify the consequences of Chagas disease control programs within the framework of decentralized health systems existing in almost all Latin America, would be crucial for the implementation and sustainability of effective and permanent epidemiological surveillance. Comparative research across countries would be useful for this purpose. More research to evaluate medium and long-term social and individual effects of preventive programs implemented in endemic countries – whether vertical or participatory - as well as the social and individual impact of control strategies in endemic and non-endemic countries is needed. Evaluating interventions using this broader perspective, and developing research tools to expand the focus of these activities to incorporate socio-cultural aspects of health is key to designing successful long-term programs. Furthermore, research focused on incorporating people's experience and needs into policies and interventions in endemic and non-endemic countries, and the development of preventive and/or control actions, conducted with attention to affected individuals beyond medical spaces, is crucial.

Some topics were scarcely addressed in the qualitative research on Chagas. For example, a transnational approach [50], which views places of origin and settlement as continuous rather than disconnected social spaces, was absent. Because migration and health research has highlighted the impact on the health knowledge and practices of the migrant's relatives in the country of origin, the study of *social remittances* [51] for health is important to understand behavior and orient interventions. Moreover, it is necessary to better understand how conceptions of Chagas disease in endemic countries – for example, resulting from the vertical implementation of prevention programs - might influence how people experience Chagas disease during migration, affecting health seeking behaviors in non-endemic countries. How infected individuals construct their identities as ill or as healthy is also particularly important with regard to understanding the mismatch between the experience of those affected and medical classifications of Chagas disease.

Further research on transdisciplinary approaches to addressing Chagas disease is required.

### Strengths and limitations of the review

The review includes papers in three languages, giving a broad overview of research conducted in different contexts, with varied health systems and at different points in the development of Chagas programs. To retrieve literature that is not electronically indexed, grey literature was also searched and incorporated. Screening was carried out in duplicate and discrepancies were solved with consensus between reviewers. Results are limited to the issues raised in the studies reviewed. Exclusion of studies whose full-text could not be accessed may be a limitation. However, because abstracts were checked, and the authors of excluded articles were included in the review through their authorship of other articles, the impact on the themes is probably minimal.

### Conclusions

Biomedical aspects of Chagas disease are embedded in socio-cultural and environmental contexts. The literature reviewed shows how qualitative social science provides key tools to understand this socio-cultural context.

This review is a potentially useful resource for policy makers, clinicians, researchers and patients. A number of findings are particularly important for the design and implementation of policies, strategies and programs. Social and structural processes are essential to explain the emergence, persistence and re-emergence of Chagas disease. However, few studies address strategies aimed at influencing the socio-structural context, and there is a general lack transferring their results in practice. Different social groups – influenced by ethnicity, socio-economic status, age, urban/rural context - experience different social conditions that influence their Chagas-related experiences and behaviors. Understanding how socio-cultural processes differentially affect these groups is key to designing and promoting appropriate interventions, adapted to populations and contexts, and considering their specific needs. Behavioral change approaches should consider how social conditions and the local representations of health and disease influence the practices related to Chagas.

Chagas disease requires an explicitly multidimensional approach, in which prevention, control and care strategies and programs are designed and implemented jointly, and in which the social and biomedical sciences, together with the experience of those affected, are incorporated and articulated.

## Supporting Information

Appendix S1 List of documents, location of data and main topic. Endemic countries.
(TIFF)

Appendix S2 List of documents, location of data and main topic. Non endemic countries.
(TIFF)

Checklist S1 Prisma checklist.
(DOC)
