# Supplementary figures and images for: Socio-Cultural Aspects of Chagas Disease: A Systematic Review of Qualitative Research
Source: PLoS Negl Trop Dis. 2013 Sep 12;7(9):e2410. doi: 10.1371/journal.pntd.0002410 (PMC3772024; doi:10.1371/journal.pntd.0002410)

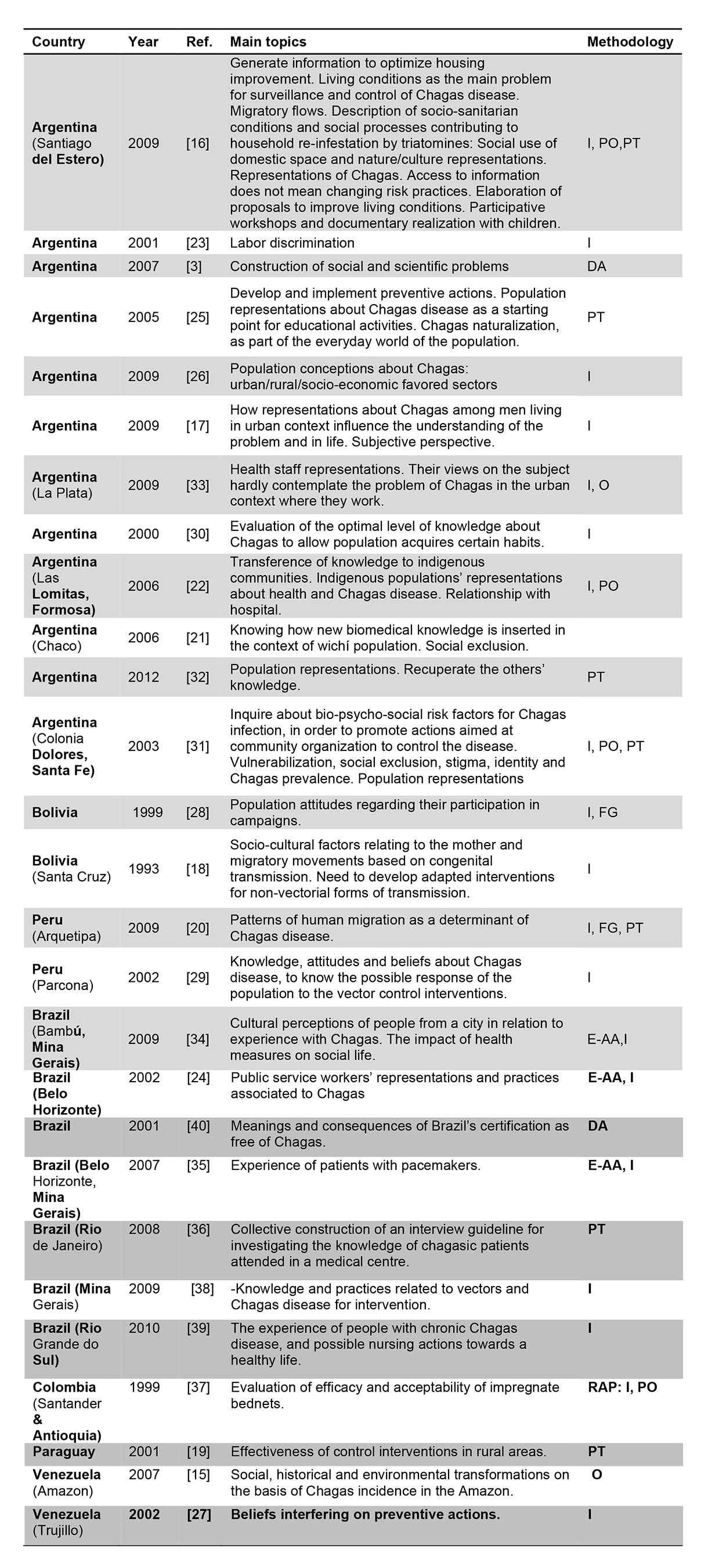

Supplement: Appendix S1 — List of documents, location of data and main topic. Endemic countries. (TIFF) [file pntd.0002410.s001.tif]

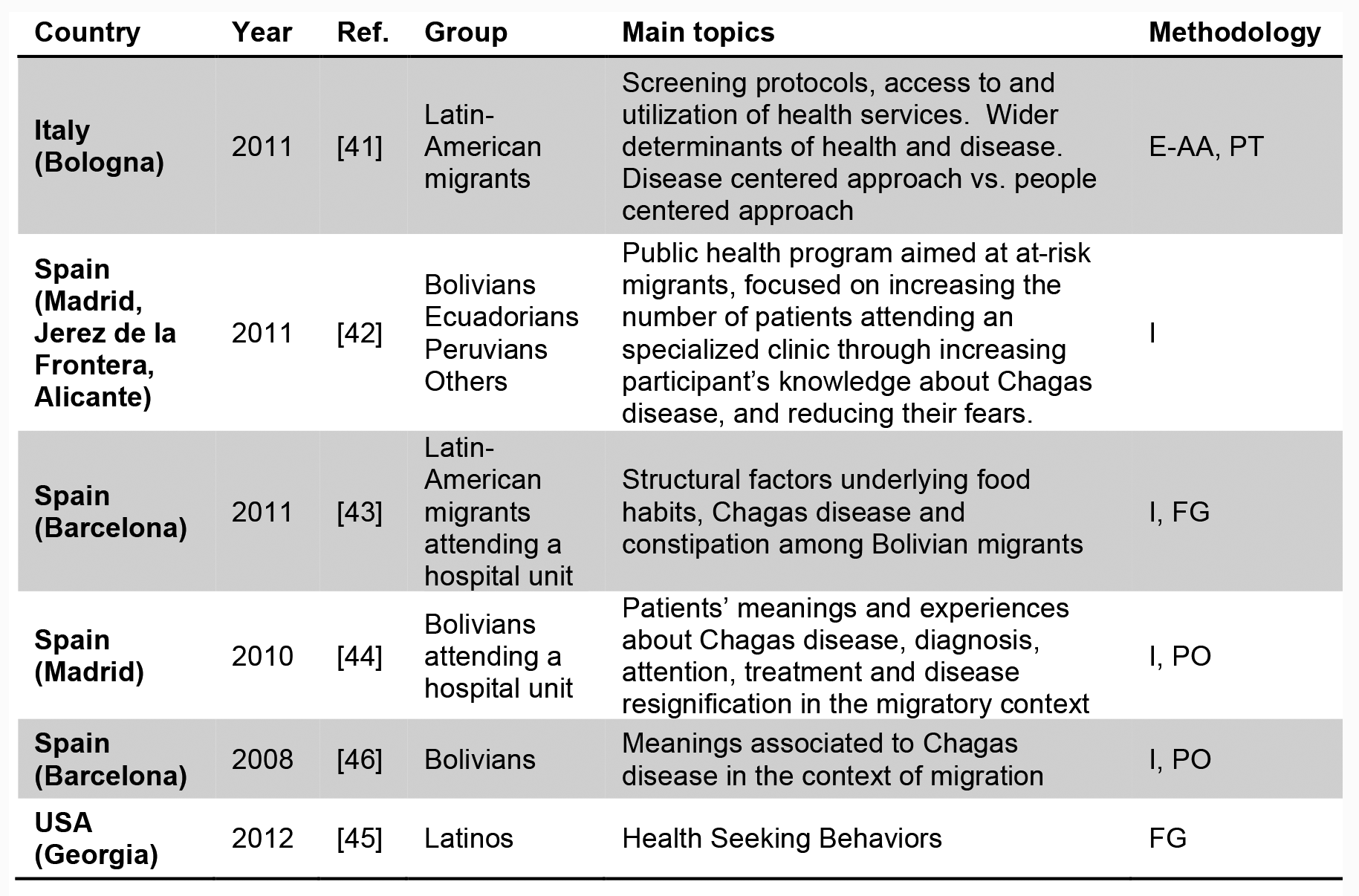

Supplement: Appendix S2 — List of documents, location of data and main topic. Non endemic countries. (TIFF) [file pntd.0002410.s002.tif]
